# The Civil Dispensaries of the Police Hospitals of Burma

**Published:** 1900-08-25

**Authors:** 


### THE CIVIL DISPENSARIES OF THE POLICE HOSPITALS OF BURMA.

In the triennial report on the civil dispensaries and police hospitals of Burma for 1896-1898 the incidence of the various diseases is discussed in detail. Malarial fevers continue to head the list. The proportion of cases to the total number of cures appears to be similar to that prevailing in other parts of India, namely, 15 to 17 per cent. Small-pox was more prevalent in the period than in the years 1893 to 1895, the number of cases being 2,090, as against 730. The question whether the spread of the disease can be traced to the immigration of coolies from Madras, and if so, whether the evil can be remedied, is still under consideration. Cholera was especially active in 1897, but the aggregate number of cases was about the same as in the previous triennium. A feature at the Rangoon Hospital has been the large proportion of tubercle and leprosy cases treated, as well as the increased prevalence in Rangoon of beri-beri. It is satisfactory to learn that the benefits of surgery are more widely appreciated. The operations performed in the three years were 48,911, as compared with 38,468 in the previous triennium. Turning to general statistics, the number of dispensaries in the province has increased from 95 to 105, and the number of civil patients from 1,706,914 to 2,296,570. Of the 99,122 in-patients, 73 per cent. were cured, or 1 per cent. less than in the previous three years. Nearly three-fifths of the patients treated were Burmese. The number of beds available has risen from 2,126 to 2,527; the daily average attendance from 1,268 to 1,405. As to the cost, there has been an increase from 4.39,056 to 4.99,447 rupees, the Government contribution being apparently materially larger owing to a rearrangement of accounts. The subscriptions from Europeans and from natives alike show an increase, and the amounts received as donations have also improved. It is pointed out in the report that some committees "appear still to have a difficulty in making up their minds to utilise the subscription funds," and inspecting officers are, therefore, recommended to advise them. With regard to the police hospitals, the number has fallen from 92 to 83. The Ruby Mines, Upper Chinderin, and Lashio Battalions are said to be the most unhealthy. It is noticeable that the cost in 1898 per thousand patients on establishment diet and medicines was considerably higher than in the case of patients treated in civic hospitals; and the difference is attributed to the large number of small detachments, for all of which separate arrangements have to be made. The expenditure on this is due to the fact that the sepoys diet themselves, while the heavy charge on account of medicines is the result of expensive field equipments. The appendices include an interesting report of the General Hospital at Rangoon, from which, however, it appears that several defects require to be remedied. There is no hot-water service laid on in the hospital; the sweepers' quarters are in a very dilapidated condition; the inside of the hospital has not been painted for seven years; the Röntgen ray apparatus has got out of repair, and has not been used "as yet;" and the tents which form the contagious diseases hospital are inadequate, and "require thorough renewal in every shape."

